# Voice Biomarkers as Possible Indicators of Cognitive Aging

**DOI:** 10.1093/geroni/igab046.2639

**Published:** 2021-12-17

**Authors:** Elizabeth Mahon, Margie Lachman

**Affiliations:** 1 Brandeis University, Waltham, Massachusetts, United States; 2 Brandeis University, Brandeis University, Massachusetts, United States

## Abstract

There is emerging evidence that measures of voice prosody are related to diagnoses of Alzheimer’s Disease and Related Disorders. The goal of this study was to examine whether voice prosody measures (pitch, pulse, voice breaks, jitter, shimmer, and amplitude) are also related to individual differences in normal cognitive aging. Data are from the Midlife in the United States Wave 2 (M2) and Wave 3 (M3) for 2693 participants (ages 42-92 at M3) who completed the M2 and M3 Brief Test of Adult Cognition by Telephone (BTACT) and had M3 voice recordings. Voice variables were measured from cognitive interviews using three cognitive tests and averaged to create a composite for each voice variable. Voice prosody was related to age, sex, education, and health, which were included as covariates. Older adults, men, and those with more health conditions had higher jitter and shimmer. Older adults, women, and those with higher education and better health had more voice breaks. Hierarchical regression models, controlling for the covariates, examined the voice composites as predictors of each cognitive measure at M3 and change over 9 years from M2 to M3. As hypothesized, higher jitter predicted lower performance and greater decline on memory, category fluency, and attention. Contrary to predictions, a lower number of voice breaks predicted worse performance and greater declines on all cognitive tests. The results suggest that voice biomarkers are related to cognitive performance and decline, and they may offer a promising approach for identifying early signs of cognitive impairment or dementia.

